# Mapping wild vascular plant species diversity in urban areas in California using crowdsourcing data by regression kriging: Examining socioeconomic disparities

**DOI:** 10.1016/j.scitotenv.2023.166995

**Published:** 2023-09-16

**Authors:** Mengyi Li, Shahir Masri, Chun-Huo Chiu, Yi Sun, Jun Wu

**Affiliations:** a Department of Disease Prevention, Program in Public Health, University of California, Irvine, CA, USA; b Department of Environmental and Occupational Health, Program in Public Health, University of California, Irvine, CA, USA; c Department of Agronomy, National Taiwan University, Taipei, Taiwan; d Institute of Medical Information, Chinese Academy of Medical Sciences and Peking Union Medical College, Beijing, China

**Keywords:** Urban plant biodiversity, Vascular plant species diversity map, Socio-economic status, Citizen science

## Abstract

Biodiversity is crucial for human health, but previous methods of measuring biodiversity require intensive resources and have other limitations. Crowdsourced datasets from citizen scientists offer a cost-effective solution for characterizing biodiversity on a large spatial scale. This study has two aims: 1) to generate fine-resolution plant species diversity maps in California urban areas using crowdsourced data and extrapolation methods; and 2) to examine their associations with sociodemographic factors and identify subpopulations with low biodiversity exposure. We used iNaturalist observations from 2019 to 2022 to calculate species diversity metrics by exploring the sampling completeness in a 5 × 5-km^2^ grid and then computing species diversity metrics for grid cells with at least 80 % sample completeness (841 out of 4755 grid cells). A generalized additive model with ordinary kriging (GAM OK) provided moderately reliable estimates, with correlations of 0.64–0.66 between observed and extrapolated metrics, relative mean absolute errors of 21 %–23 %, and relative root mean squared errors of 27 %–30 % for grid cells with ≥80 % sample completeness from 10-fold cross-validation. GAM OK was further applied to extrapolate species diversity metrics from saturated grid cells (*N* = 841) to the remaining grid cells with <80 % sample completeness (*N* = 3914) and generate diversity maps that cover the grid. Further, generalized linear mixed models were used to examine the associations between species diversity and sociodemographic indicators at census tract level. The wild vascular plant species diversity metrics were inversely associated with neighborhood socioeconomic status (i.e., unemployment, linguistic isolation, educational attainment, and poverty rate). Minority populations (i.e., African American, Asian American, and Hispanic) and children had significantly lower diversity exposure in their neighborhoods. Crowdsourcing data offers a cost- effective solution for characterizing large-scale biodiversity in urban areas.

## GRAPHICAL ABSTRACT

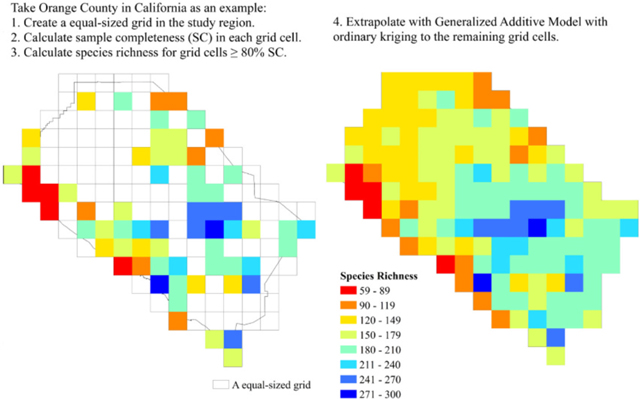

## 1. Introduction

Decades of studies have shown the impact of biodiversity loss on the dynamics, functioning, and services of ecosystems, which in turn undermines the capacity of ecosystems to provide goods and services for humanity (Aerts et al., 2018; Andersen et al., 2021; Cardinale et al., 2012). Diverse plant communities provide more resilience to both human and ecosystem health (Shroff and Cortés, 2020 ). In recent years, there is a growing body of research that has shown the beneficial impacts of exposure to nature, green space, and biodiversity on human health, including physical and mental aspects (Aerts et al., 2018; Andersen et al., 2021; Marselle et al., 2021).

The attention restoration theory (Kaplan and Kaplan, 1989) and stress recovery theory (Berto, 2014) suggest that natural environmental experience can enable recovery of mental fatigue and concentration. Individuals who spend time in biodiverse environments have reported feeling a sense of “being away,” which helps improve mood and restore the capacity to concentrate (Hedin et al., 2022; Roswell et al., 2021). In addition, the biodiversity hypothesis (Haahtela, 2019) postulates that early-life exposure to diverse microbial environments modulates and diversifies human commensal microbiota, which trains the immune system to promote immune tolerance and thus protect against allergies and inflammatory disorders. By exploring biodiversity, we can gain a deeper understanding as to how nature and the environment affect public health.

Biodiversity encompasses a wide range of factors, including species, genetics and ecosystem diversity, and can be measured by biotic communities/processes, the number of unique species, the amount (e.g., abundance, cover, biomass) and structure of each species, and the habitat in which such species live (DeLong, 1996). The terms “species richness” and “diversity” are often used to indicate the variety of organisms within a region, and are the most intuitive and fundamental indicators of biodiversity (Colwell and Coddington, 1994).

Direct observation and identification, transect sampling, and plot-based surveys are the most prevalent approaches (Aerts et al., 2018) that measure species diversity in environmental health studies. While these methods provide thorough and high-quality diversity measurements in the sampled region, they require intensive sampling and skilled researchers, making such methods only practical over small areas (e.g., neighborhoods, urban parks) and with experienced research teams. Moreover, these methods are usually tailored to address a specific research question such as examining the distribution of a particular set of species. The results of such efforts are also disconnected and contain varying data elements that are not readily harmonized to enable continuous mapping of biodiversity across large areas.

The introduction of satellite imagery has greatly enhanced efficiency in data collection and allowed the mapping of species diversity over large areas, but it is expensive to apply and requires extensive labor to conduct botanical field surveys that can predict species diversity distributions (Yang et al., 2022). Additionally, since satellites take aerial images, they cannot assess species diversity below tree canopies (Bae et al., 2019). Further, complex urban features can interfere with such data collection (e.g., plant detection obscured by bridges, buildings, or shadows), making it challenging to characterize plant diversity in urban areas.

Recently, citizen scientist-gathered databases (e.g., iNaturalist), also known as crowdsourced data, have been increasingly used in plant taxonomic diversity-related health research (Donovan et al., 2021a, 2021b) and have been shown to be of comparable quality as professionally gathered data (e.g., national Conservation Data Center databases) (Roman et al., 2017). While crowdsourced data is a cost-effective way to provide biodiversity information across wide spatial scales, current research does not fully account for the important issues of geographic bias and user selection bias caused by the non-random nature of data collected by citizen scientists (Donovan et al., 2021a, 2021b).

Geographic selection bias may be introduced by landscaped areas in which certain areas are accessible and others impenetrable, while user selection bias may be caused by the spatially nonrandom distribution of the data collectors themselves. In addition, those collecting data may disproportionally submit observations of socially popular species, thus causing unequal sampling spatially (Uyeda et al., 2020). If not addressed, biased sampling can lead to biased estimation of biodiversity, and result in biased or false conclusions in health studies. To reduce the geographic and user selection bias, we focus on urban areas. These regions are readily accessible by large populations and host many bioblitzes led by the local natural history museums or conservation organizations such as City Nature Challenge. Bioblitzes are a widely used type of citizen science event in which scientists, naturalists, and the public work together to record and identify many species within a certain period and region (Roger and Klistorner, 2016). Therefore, urban areas may have more records of species that extend beyond socially popular species. To correct the problem of unequal sampling efforts across areas, we applied a sample completeness-based approach (Chao and Jost, 2012) that enabled us to identify areas with saturated observations (mostly in metropolitan cities) and derive species diversity metrics accordingly. However, such methods cannot be used for areas with unsaturated observations (mostly in less populous urban areas) where the number of records is low. Following our spatial characterization of species diversity using crowdsourced data, we employed spatial methods such as ordinary kriging and regression kriging to help fill in the areas with unsaturated sampling data.

Biodiversity may be unevenly distributed across subpopulations. Income level and race/ethnicity are reported as important indicators of access to areas with a high level of biodiversity and green space in the United States (Lin et al., 2021; Sun et al., 2021). Specifically, previous studies have shown positive associations between plant species diversity and income (Blanchette et al., 2021; Kuras et al., 2020). In addition, better understanding of the relationship between diversity and age is important, especially considering the potentially beneficial role of biodiversity in early human development, as suggested by the Biodiversity Hypothesis (Haahtela, 2019) and other studies (Cavaleiro Rufo et al., 2021, 2020; Marselle et al., 2021; Winnicki et al., 2022). However, few studies have examined species diversity inequalities across socioeconomic dimensions, such as educational attainment, unemployment, race/ethnicity, and age.

In this study, we developed an analytic framework to address three major challenges (i.e., geographic selection bias, user selection bias and unequal sampling bias) in using crowdsourced data to characterize species diversity levels. After completing mapping, we further investigated census tract (a geographic unit)-level diversity metrics in relation to socioeconomic factors, as well as vulnerable population indicators.

Specifically, we aimed to understand: 1) whether crowdsourcing data can estimate species diversity at a fine spatial resolution (5 × 5-km^2^ resolution); 2) which method is the most appropriate for estimating diversity metrics in areas with low sampling completeness; 3) whether species diversity is correlated with population SES and vulnerable population indicators and, if so, which groups are disproportionately affected.

## 2. Methods

### 2.1. Wild plant data source and study area

We obtained research-grade records of observed wild vascular plant species from the iNaturalist platform available through the Global Biodiversity Information Facility. This platform is a global data repository for museum specimen records and citizen science observations. The research-grade data in iNaturalist is free and publicly accessible, and is accompanied by accurate geographic coordinates (Uyeda et al., 2020) and taxonomic identifiers (Hart et al., 2023) that have been increasingly shown to support reliable mapping of taxonomic diversity (Callaghan et al., 2022; E. Li et al., 2019). The iNaturalist platform was founded in 2008, with its data repository having dramatically increased since 2013. In 2019–2022, there were over 300,000 research-grade observations recorded in California annually.

To minimize temporal biases caused by higher rates of recording during warmer seasons, weekends, and holidays, we utilized the most recent four years of data between January 1st, 2019 and December 31st, 2022 (GBIF.org, 2023). Although iNaturalist allows extensive observation recording, research-grade observations are defined by iNaturalist as those containing photos and dates, georeferences, and relate to naturally growing (as opposed to cultivated) species.

In this study, we focused on examining species diversity (henceforth “diversity”) of wild vascular plants, which is a strong indicator of total biodiversity across environmental gradients and broad taxonomic realms (Brunbjerg et al., 2018) in urban areas of California, United States. To accomplish this, we first separated urban census tracts from rural census tracts based on 2010 rural-urban commuting area codes (Morrill et al., 2010), which use population density, urbanization, and daily commuting measures to delineate rural and urban areas in the US. Areas with a primary code of 1.0, which indicates a metropolitan area core, were defined as urban in our study.

Second, we created a grid comprised of equal-sized 5 × 5-km^2^ squares (*N* = 4755) that covered the entire study region. Compared to the use of administrative units (e.g., census tract), the use of a uniform grid enables the removal of bias caused by high numbers of records over a larger sampling area (Gotelli and Colwell, 2001). Importantly, only grid cells with observations reported by a minimum of 40 unique citizen science participants (each participant may upload one or more entries of plant species, with an average of 20 records per person) were included in the initial analysis. This inclusion criterion was based on an assessment of citizen scientist-collected data (Callaghan et al., 2022), which found an average of 44 completed bird reports made by citizen science participants while walking in a certain area were needed to meet 95 % sample completeness in a 5 × 5-km^2^ grid cell. We further cleaned the data by removing incomplete observation records (i.e., records that were missing taxonomic information or geographic coordinates). Since individual species usually present a spatial aggregation pattern, we converted the abundance data to incidences, which better fit our model assumptions of random sampling (Chiu, 2022).

### 2.2. Plant species diversity indices

To reliably infer true species diversity from crowdsourcing data, we used the unified framework of Hill numbers, which incorporates three widely used measures of biodiversity: species richness (order q = 0), Shannon diversity (the exponential of Shannon entropy, q = 1), and Simpson diversity (the inverse of Simpson concentration, q = 2). This methodology enables the differential weighing of rare species (the higher the order, the more sensitive the method is to rare species). Output diversity estimates were expressed in units of numbers of species, which has advantages in probing the complexity of biodiversity within species communities (Hill, 1973). Although these three indices tend to be highly correlated, they provide a comprehensive picture of biodiversity and are therefore recommended for combined use (Roswell et al., 2021).

**Fig. 1.**
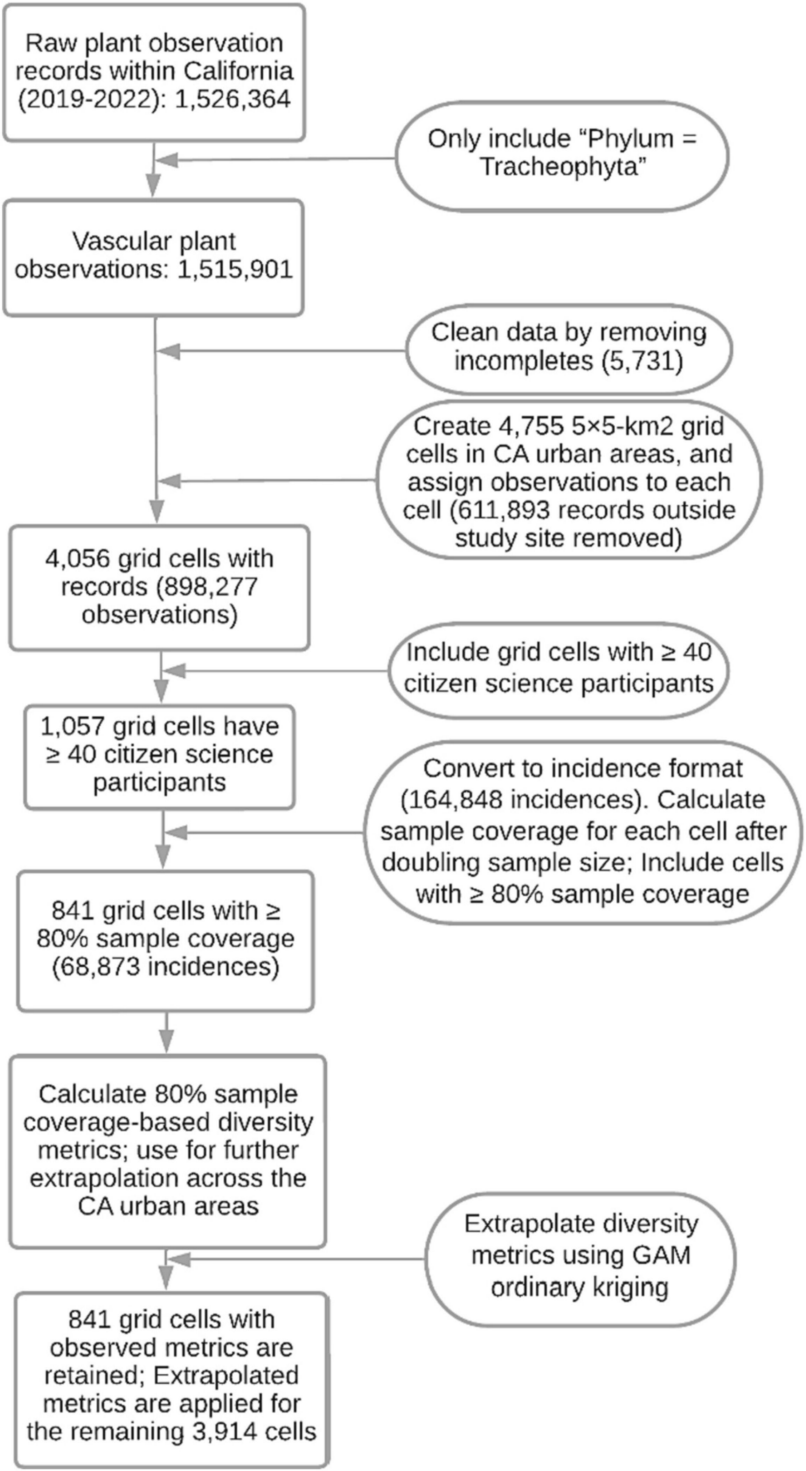
Procedures for data cleaning and diversity metrics development.

The sample coverage/completeness describes individuals in the community that belong to the species captured by sampling (Chao and Jost, 2012). With this method, we can effectively identify areas with saturated observations and calculate species diversity metrics by a fixed sampling coverage. First, we doubled the sample size of the observations in each grid cell to calculate sample coverage. By doubling the sample size and examining the resulting species accumulation curve, we were able to assess the adequacy of sampling completeness in each grid cell (Chao et al., 2020). In this study, the correlation between 80 % sample completeness and 90 % sample completeness for species richness, Shannon diversity, and Simpson diversity were 0.96, 0.98, and 1.0, respectively, indicating that the use of 80 % completeness criterion would adequately capture the grid cells with saturated sampling. Thus, we defined saturated grid cells as those with at least 80 % of sampling completeness, which maximized the number of saturated grid cells while allowing for valid comparisons across these grid cells. We further calculated the diversity estimates at 80 % sample completeness for each saturated grid cells and then applied kriging and regression kriging to extrapolate diversity metrics for unsaturated grid cells. All computations of sampling coverage and diversity estimates for saturated grid cells were conducted using the R package *iNEXT* (Hsieh et al., 2016). Fig. 1 shows the flowchart of the procedures for data cleaning and diversity metrics development.

### 2.3. Spatial modeling, extrapolation and validation

In order to extrapolate species diversity over geographic areas with unsaturated sampling data, we first tested 27 variables as potential explanatory variables (see Table S1 for full list of all examined variables and their references) for use in vascular plant species diversity modeling. Such variables were those related to water-energy dynamics, vegetation indices, tree and land cover, road density, and soil properties. These variables were chosen due to their proposed relationships with diversity. That is, the water-energy dynamics hypothesis proposes that the availability of water and energy resources shapes habitat suitability and can explain patterns of species diversity (Kreft and Jetz, 2007). Water-energy dynamics variables tested in our model were precipitation, temperature, elevation, actual evapotranspiration, and potential evapotranspiration, which have been reported as strong predictors for vascular plant species diversity (Chaudhary et al., 2021; Kreft and Jetz, 2007). Similarly, urban greenspaces and landscapes have been shown to harbor a greater richness of vascular plant species (Labadessa and Ancillotto, 2023; MacGregor-Fors et al., 2016). In addition, anthropogenic features such as land cover and road density have been identified as major predictors of plant species richness in urban settings (Aronson et al., 2014; Beninde et al., 2015; Godefroid and Koedam, 2007). Lastly, soil properties can also explain the taxonomic diversity of vascular plants (Cheng et al., 2018).

Precipitation and temperature were derived from a historical annual average dataset. Elevation was obtained from 30 m resolution 2010 Global Multi-resolution Terrain Elevation Data. Actual evapotranspiration data was obtained from the Basin Characterization Model with a resolution of 270 m (Flint and Flint, 2012) whereas potential evapotranspiration data was obtained from Zomer et al.’s (2022) work. To assess greenspace and other land features, we examined the mean normalized difference vegetation index (NDVI) and percentages of tree canopy, each land cover type, and four road types within each spatial grid cell. The 4-year average annual NDVI, which measures the vegetation density on the ground, was generated from Terra (MOD13Q1) satellite products from NASA, with a spatial resolution of 250 × 250-m^2^. Raster data on tree canopy, land cover and road density at a resolution of 30 × 30-m^2^ were downloaded from the 2019 National Land Cover Database (NLCD). Land cover measurements within each spatial grid cell were quantified by computing the proportion of the area covered by water, developed open space, forest, herbaceous vegetation, planted/cultivated cover, and wetlands areas. Road density, including primary, secondary, tertiary and thinned roads, were extracted from the NLCD 2019 Developed Imperviousness Descriptor Database (Dewitz, 2021).

Soil data was obtained at an 800 m resolution from the Soil Survey Geographic Database (SSURGO) (Soil Survey Staff) which provides standardized soil properties information of soil calcium carbonate content, cation exchange capacity, electrical conductivity, soil pH, sodium adsorption ratio, soil organic matter, available water holding capacity and bulk density. The soil survey data is a continually updated source from which we extracted average values for each grid cell. In this study, most soil data was collected in 2022, while a minority came from earlier years. In instances where the SSURGO database contained missing values for soil properties, we employed a spatial interpolation approach to estimate these values. Initially, we tested radii of 6 km, 7 km, and 8 km from the centroid of each grid cell to determine the optimal distance for interpolating the missing data. It was found that an 8 km radius was sufficient to assign new values to all grid cells with missing data. Consequently, we utilized an 8 km radius to extract mean values from the SSURGO dataset for these areas. Grid cells with existing data were retained and used as-is, ensuring the most accurate representation of soil properties across the study area. Since the latitudinal gradients in vascular plant diversity have been extensively examined in previous research (Chaudhary et al., 2021; Sabatini et al., 2022), the coordinates of centroids of each grid cell were also included as predictors.

Next, we extrapolated the diversity metrics in saturated sampling grid cells to unsaturated sampling locations of the study area using common geostatistical methods: ordinary kriging (OK; spatial interpolation only with autocorrelation considered but no new information from covariates) and four regression models with covariates, a generalized linear model (GLM) with and without OK, and a generalized additive model (GAM) with and without OK. These five models were compared and the method with the best performance was selected to generate species diversity maps. For the four regression models with and without OK, all co-variables were standardized to have a mean of 0 and standard deviation of 1. Pairwise Pearson’s correlation tests were subsequently conducted. In cases where two predictors were collinear (*r* > 0.8), we removed the one with the lower outcome (diversity metrics) correlation. Latitude was not included in the GLM analysis due to collinearity with longitude yet was added to the GAM analysis and was modeled with longitude as a two-dimensional smoothing term with Dunon splines to account for spatial autocorrelation.

The *mass* and *mgcv* packages were applied, respectively, to investigate the linear (GLM) and non-linear (GAM) relationships between the diversity metrics and co-variables. We fit the models with a quasi-Poisson distribution, which is considered an appropriate tool for the analysis of overdispersed species count data (Ver Hoef and Boveng, 2007). The predictors with >0.1 correlation with diversity metrics were retained and subjected to stepwise variable selection procedures. The model construction was assessed using the proportion of deviance explained, which measures the total variability captured by the model. In order to avoid overfitting, Restricted Maximum Likelihood and double penalty approaches were employed using the *gam* function of *mgcv* in R (Marra and Wood, 2011). We then kriged the residuals from the regression models, which represented the stochastic factors, by fitting the optimum variogram. The residual variations from kriging were then superimposed to the regression results as diversity metrics.

To assess the performance of the five models (OK, GLM, GAM, GLM OK, and GAM OK), we conducted 10-fold cross-validation. That is, 90 % of the training data for each model was randomly selected for model development and optimum variogram fitting, and the remaining data was used for validation. The procedure was then repeated ten times, using a new 10 % held-out subset of data each time. The correlation between the observed and extrapolated diversity metrics, deviance explained, mean absolute error (MAE), relative mean absolute error (RMAE), root mean squared error (RMSE), and relative root mean squared error (RRMSE) were obtained from the validation procedure and examined to confirm the robustness of the model. These values were also used to compare geostatistical techniques to determine the most reliable and reasonable method for extrapolation. In using the four error measures (MAE, RMAE, RMSE, and RRMSE), lower values indicate better extrapolation performance.

Finally, to optimize the final maps of diversity metrics, we made modifications to the results from the “best” model selected from the previous step. We retained the extrapolated values in the unsaturated grid cells and replaced the metrics in saturated grid cells with the observed estimates, which was referred to as the optimized version.

### 2.4. Uncertainty

We calculated standard errors of predictions from GAM OK to reflect the uncertainty. The uncertainty maps show areas with more or less confidence in prediction and highlight the areas that need more data collection from citizen science (Jansen et al., 2022).

### 2.5. Association of diversity with socioeconomic factors

After the optimal species diversity maps were generated from the work described above, we extracted the mean diversity metrics from the 5 × 5-km^2^ grid to each census tract. Sociodemographic factors were retrieved from the CalEnviroScreen4.0 tool (CES4.0) (OEHHA, 2021) that was developed by the California Environmental Protection Agency (CalEPA) and its Office of Environmental Health Hazard Assessment to provide census tract-level data on environmental health, public health, and SES conditions throughout the state. In the U.S., census tracts generally have a population size between 1200 and 8000 people, with an optimum size of 4000 people with similar population characteristics, economic status, and living conditions (U. S. Census Bureau, 2022). In our study region, the spatial size of census tracts (10.03 ± 65.42 km^2^) varies widely depending on the population density. In this study, we examined five neighborhood SES factors (educational attainment, housing burden, linguistic isolation, poverty, and unemployment), a composite population characteristics score [average percentile for three sensitive population indicators (asthma, cardiovascular disease, and low birth weight) and five SES factors], race/ethnicity (Hispanic, non-Hispanic White, African American, Native American, Asian American, and Multiple races), two age groups (children <10 years and elderly >64 years), and the overall CES4.0 score (multiplication of the pollution burden and population characteristics scores). In order to ensure comparability across variables, we rescaled the population characteristics score from their original range of 0–10 to a 0–100 scale. Detailed information is shown in Table S2. Notably, CalEPA identified census tracts with the highest 25 % of the CES4.0 scores or the highest 5 % of CES4.0 cumulative pollution burden scores as disadvantaged communities (DACs) based on Senate Bill 535 (CalEPA, 2022). In total, there were 2155 DACs and 4984 non-DACs in the study region.

We examined the correlation of our mean plant diversity metrics with SES, population characteristics scores, race/ethnicity, and vulnerable population indicators at the census tract level using Pearson’s correlation coefficients. Additionally, a *t*-test was employed to determine whether statistically significant (*p*-value <0.05) differences existed between diversity metrics averaged across DACs and non-DACs.

To reduce the potential influence from population density and spatial clustering, we employed generalized linear mixed models (GLMMs) with normal distributions to further examine associations between our model-estimated species diversity metrics and sociodemographic variables at a continuous scale. All models included one of the sociodemographic variables as the main fixed effect and adjusted for population density. We chose the exponential spatial covariance structure to account for spatial autocorrelation in the diversity metrics outcome and used “county” as the random effect. We also did two sensitivity analyses: 1) included all SES factors as a full model, and 2) added NDVI as a confounder in the models. In the full models, the poverty and the housing burden variables were excluded to avoid collinearity. We then employed backward selection and evaluated both AIC and BIC as criteria in the full models with three SES variables (education attainment, linguistic isolation, and unemployment). All geostatistical analyses and mapping procedures were conducted using R 4.1.3 and ArcMap 10.8.2, while GLMMs were performed using SAS 9.4 (SAS Institute, Inc., Cary, NC).

## 3. Results

### 3.1. Urban plant diversity characterization and results from sampling completeness analyses

Based on the 2019–2022 records from our study region, the most prevalent observations were those of the flowering plant (Angiospermae) subphylum, which contained dicot (3484 species among 583,007 records) and monocot (675 species among 76,142 records) classes, followed by 61 species from the fern class (14,654 records), 51 species of conifers, and <12 species of lycophytes, gnetophytes, and ginkgos (See Appendix A for full species list).

**Fig. 2.**
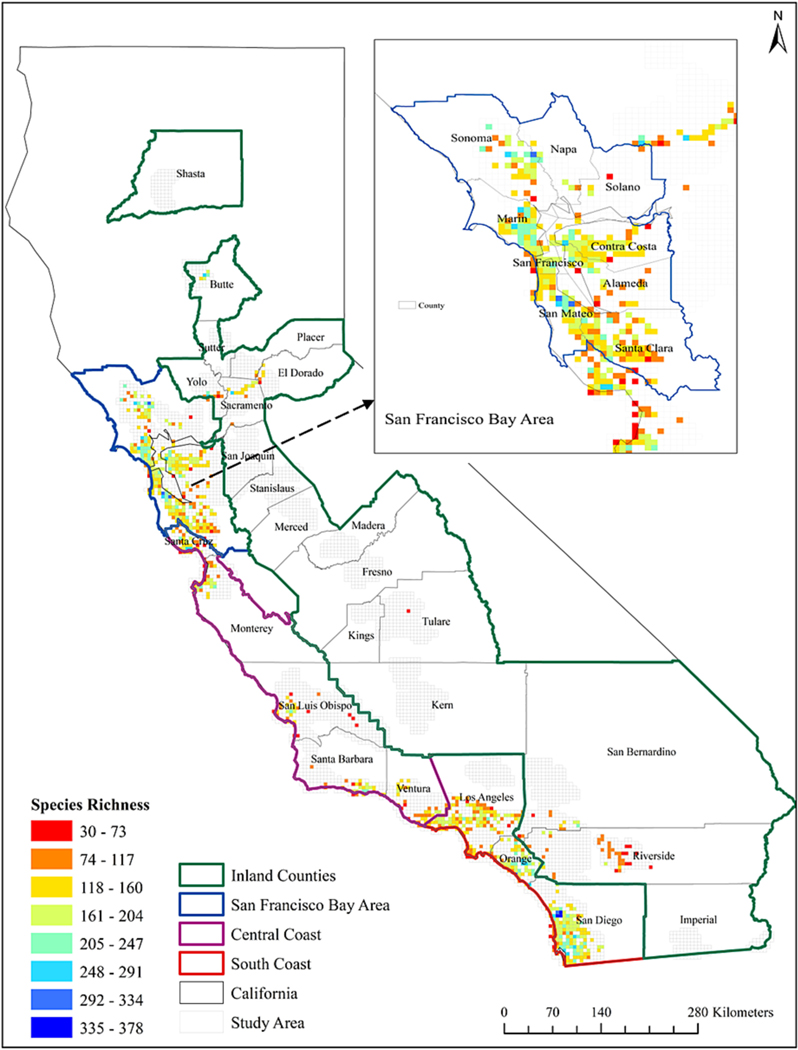
The 80 % sample coverage-based wild vascular plant species richness metrics within the study region.

Roughly 17.7 % of grid cells (841 out of 4755) had an estimated sampling coverage >80 %, which included 3757 vascular plant species (See Appendix B for full species list). Summary statistics of plant diversity metrics for 841 saturated sampling grid cells are presented in Table 1. Saturated sampling grid cells (Fig. 2) were mostly located in northern and southern coastal areas with high population densities, including Marin, San Francisco, Santa Mateo, Los Angeles, Orange, and San Diego. Areas located in the Central Coast and the inland areas such as San Joaquin, Stanislaus, Merced, Madera, Tulare, and Kern had few or no saturated grid cells for extrapolation.

### 3.2. Diversity metrics extrapolation and final maps

GAM OK slightly outperformed GAM and OK in extrapolating diversity metrics. Validation results in Table 2 show that GAM OK had the lowest RMAE (21 %–23 %) and RRMSE (27 %–30 %), and the highest correlations (0.64–0.66) between the observed metrics with the extrapolated ones for three diversity indices. Thus, GAM OK was used to predict diversity in unsaturated grid cells. The included co-variables and details for GLM and GAM are shown in Table S3. The statistics of the observed diversity metrics, GAM OK extrapolated values and the final optimal maps, including both saturated grid cells (observations) and unsaturated grid cells (modeled), are presented in Table 1.

**Fig. 3.**
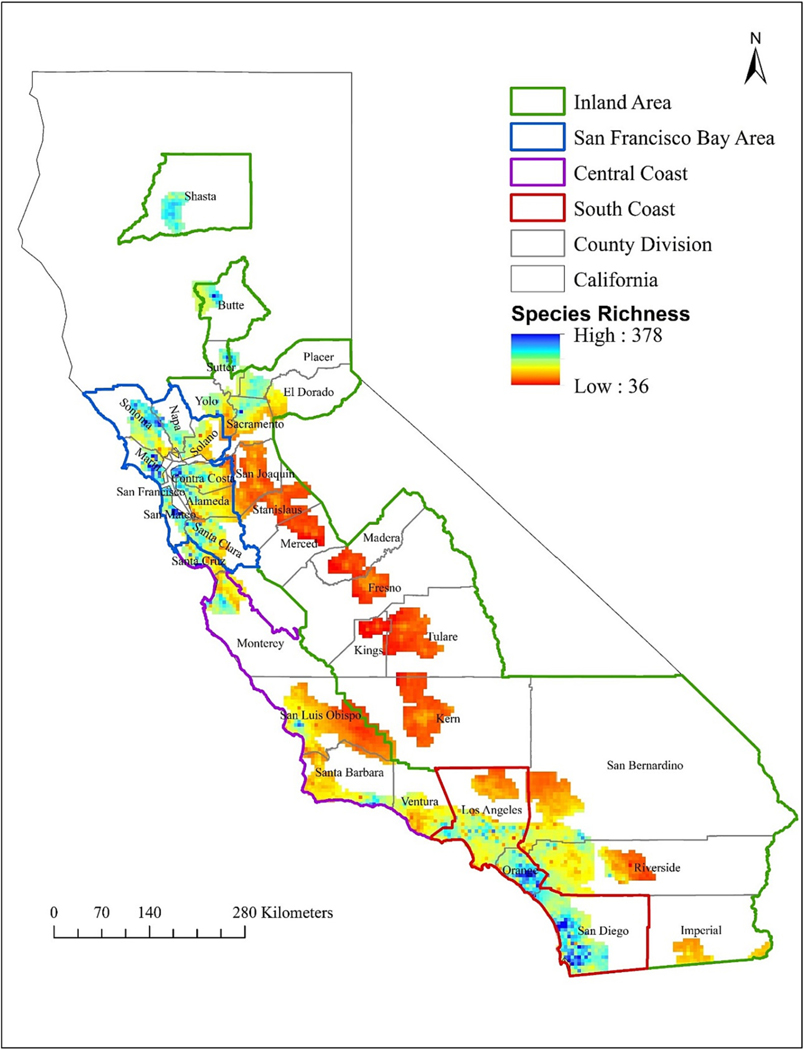
Spatial distribution of wild vascular plant species richness metrics in California urban areas (2019–2022) (resolution: 5 km). Notes: For visual purposes, we cut off the grid cells outside the California land boundary. This resulted in a discrepancy in the minimums when comparing Fig. 2 with the optimized version of metrics in Table 1.

Three diversity metrics are highly correlated (correlation coefficients range from 0.95 to 0.98) and show similar distribution patterns. We took species richness metrics as an illustration and depicted their spatial distribution across California urban areas in Fig. 3. Low plant species diversity patterns appeared in inland counties where few to no saturated sample sites existed. Yet, the northern region of the inland counties, for example, Shasta, Butte, and Sutter, had higher species richness. The counties located alongside coastal areas tended to harbor more species diversity hotspots, compared to inland counties. Notably, in the southern coast, the diversity in Los Angeles County was at middle to low levels, while Orange County and San Diego County had higher diversity values. The prediction uncertainties were higher in the inland counties than those in the coastal areas (Fig. S3).

### 3.3. Census tract-level diversity metrics and its association with sociodemographic indicators

The diversity metrics were significantly lower in DACs than in non-DACs (*p* < 0.001) (Table 3). The characterization of population characteristics score, SES factors, race/ethnicity, and vulnerable population indicators are depicted in Table S4. After controlling for population density and spatial clustering (Table 4), we found that four SES factors were significantly inversely related to diversity metrics, with associations being most pronounced for unemployment rate (e.g., species richness: −0.61, 95 % CI: −0.81, −0.4), followed by linguistic isolation, educational attainment, and poverty rate. The proportion of children had a negative association with diversity metrics (e.g., species richness: −0.79, 95 % CI: −0.96, −0.62). Hispanic, African American, and Asian American races were significantly negatively correlated with diversity metrics; while non-Hispanic Whites, and residents of mixed race were found to be significantly positively correlated with diversity metrics. Overall, for a one unit increase in the standardized population characteristics score (0–100, higher score indicates greater vulnerability), the species richness metrics decreased by 0.22 (95 % CI: −0.26, −0.18).

Compared to the models with a single SES variable, the full models (Table S6) showed similar or moderately attenuated associations between species diversity and SES factors, including educational attainment, linguistic isolation, and unemployment. The significance levels between linguistic isolation and species diversity shifted from significant in the single factor models to insignificant in two full models (i.e., species richness and Simpson diversity). Sensitivity analysis that was further adjusted for NDVI (Table S7) showed slightly attenuated or similar associations between the species diversity and the sociodemographic factors.

## 4. Discussion

To our knowledge, this is the first study that depicts wild vascular plant species diversity across a state-wide area using crowdsourced data. We developed an analytic workflow, which addressed geographic and user selection bias along with issues related to unequal sampling by citizen scientists. Our approach derived diversity metrics in adequately sampled areas and achieved a moderate performance in extrapolation. Results showed wild vascular plants exhibit greater species diversity in coastal areas than inland areas. We also found lower SES and minority populations and communities with a higher percentage of children had lower species diversity levels.

This study demonstrates data gathered by citizen scientists to be a valuable source to generate proxy estimates of biodiversity and can inform studies on complex associations between biodiversity and public health. Observations from such databases include the organisms that citizens are exposed to or find noteworthy in their daily lives, thus reflecting the daily interactions between humans and nature. Furthermore, the growing body of lay people who contribute to the collection and reporting of data has enabled extensive growth in data collection without data acquisition costs (Heberling et al., 2021). In California, the number of vascular plant observations and species (reported through iNaturalist) increased 238-fold and 5-fold since 2008, respectively. In total, 6492 vascular plant species were identified in our raw dataset (2019–2022), with approximately 5000 species reported each year. This is comparable to the 6609 recognized vascular plant species reported by the Jepson Flora Project (2023), which encompasses the most comprehensive and scientifically accurate sources of California flora. Despite the advantages of crowdsourcing data, existing bias may impede its straightforward application in environmental health research.

In our analytical workflow, we explicitly incorporated sample completeness, which allowed us to identify areas with saturated observations, and to quantify the standardized species diversity levels based on community characteristics, rather than the sampling efforts (Chao and Jost, 2012). This approach offers an improvement over the point-to-grid method used in the most recent epidemiology studies (Donovan et al., 2021a, 2021b), which aggregated individual point observations into larger units (land cover classes) and then generated diversity metrics to the grid level (meshblock) to address the non-random sampling issue in citizen science data. This point-to-grid approach requires an underlying assumption that the distribution of species is closely related to land cover attributes. For instance, if more observations/species are reported in densely urban areas due to clustered citizen scientists, the results will show an urban tendency, which neglects the species distribution pattern in relation to complex ecosystem functioning and environmental variation. Our analytical framework can also be applied in other volunteer-collected species monitoring databases that contain unbalanced sampling, such as eBird and eButterfly. Furthermore, our sampling coverage profiles highlight under-sampled regions, such as California urban areas where few or no saturated grid cells were found in the Central Coast and middle inland zones, suggesting that more project initiatives led by natural history museums or similar conservation organizations are needed to fill these data gaps.

In terms of the diversity pattern across California urban regions, our results are in accordance with two assessments (Love et al., 2022; Ma et al., 2020) which found that urban plants exhibit greater species diversity in coastal areas than inland areas. This may be the result of a convergence of cultivated plants and wild-growing flora species within urban areas. That is, communities with a highly diverse urban landscape may attract a higher level of wildlife. We presume that the typical urban garden provides suitable habitat for cultivated plants and therefore may further increase wild plant diversity. The synergy between cultivated plants and wild plants was found in both plant species abundance and richness in urban environments (X.-P. Li et al., 2019; Seitz et al., 2022), implying that urban planning may facilitate the coexistence of cultivated plant diversity and wild plant diversity. However, wild species may be immediately managed as weeds, especially for lawns in urban settings (Stewart et al., 2009), leading to the reduced species richness and abundance of wild plants. The association between wild plant species richness and cultivation or maintenance intensity is inconclusive from previous studies, and needs more investigation. Overall, our results provide a visualization of urban wild biodiversity as well as uncertainty estimates, which can help urban planners in resource allocation, promoting the planting of more diverse species, as well as wildlife and biodiversity conservation.

Residents of low SES and minority groups may experience multiple different disadvantages as it relates to health and plant diversity. First, such residents are already known to suffer from health disparities with respect to social determinants (Williams et al., 2010). Secondly, given that they are more likely to live in communities with poor irrigation systems and low environmental quality, they are more likely to experience low biodiversity within their neighborhoods. The fact that low-income areas exhibit low plant species diversity is well established in previous studies (Hope et al., 2003; Leong et al., 2018). Our findings aligned with the luxury and legacy effect in biodiversity (Hope et al., 2003; Kinzig et al., 2005), which presumes a global pattern of a positive relationship between plant species diversity and affluent neighborhoods. Moreover, mixed conclusions have been drawn from previous research in more affluent communities. In the high-density and high-SES areas, negative or neutral relationships with biodiversity levels have been reported, due to limited space to increase plant diversity (Kuras et al., 2020). These results may help to explain the moderate-to-low levels of plant species diversity observed in this study in non-disadvantaged parts of the densely populated urban areas of Los Angeles County. However, previous studies focused on cultivated plants, whereas our study utilized wild plants. Thus, a direct comparison of our findings with those of previous studies is not feasible. Specifically, we found an inverse relationship between wild plant species diversity and the proportion of minority residents, such as Hispanics/Latinos, Asian Americans, and African Americans. This result is congruent with a study conducted in 268 urban locations throughout the United States, which also shows reduced genetic diversity and urban wildlife populations and attributes to be related to racial segregation in non-White neighborhoods (Schmidt and Garroway, 2022). Such results suggest a correlation between the unequal distribution of plant species diversity and social inequality.

Accumulating studies have demonstrated the positive effects of plant diversity on microbial biomass (Chen et al., 2019), soil microbial diversity (Baruch et al., 2021; Liu et al., 2020), and other animal diversity such as birds and insects (Peng et al., 2022). The biodiversity of vegetation surrounding residencies significantly influences the composition of commensal skin bacteria and airborne microbial content (Prescott et al., 2017). Early-life exposure to microbial biodiversity has been proposed to have a positive effect on the human microbiome and its immunomodulatory capacity, which can potentially protect against allergies, autoimmune diseases, and various non-communicable diseases during later life (Cavaleiro Rufo et al., 2021; Marselle et al., 2021). In a study conducted in Finland, it was found that atopic adolescents had a lower vascular plant species richness in the surroundings of their homes and a significantly reduced diversity of beneficial bacteria on their skin compared to healthy individuals (Hanski et al., 2012). In addition, due to the lack of species diversity maps and onerous field data collection, most current research only focuses on biodiversity levels over small areas, such as residential gardens or school areas. In contrast, the methods and species diversity map illustrated in this study provide heterogenous exposure levels across a large region, thus advancing our understanding of community exposure to species diversity in various settings and how it may influence human health and well-being, especially for children.

Several limitations should be noted. First, this study focused solely on species diversity and therefore cannot comprehensively capture the complexity of biodiversity. Second, the diversity metrics were based on citizen science observations and therefore may not reflect the actual biodiversity for two main reasons: 1) there could be bias in the preference of citizen science participants recording plant species with certain plant traits (e.g., aesthetics, size, rarity, conspicuousness); and 2) we calculated diversity metrics based on an 80 % sample completeness rather than a 100 % sample completeness. Thus, a field- and expert-based approach in biodiversity sampling is still necessary and pivotal to provide valuable botanical data and validate observations from citizen science datasets. Nevertheless, our proposed approach is useful to efficiently map observed biodiversity on large scales, as well as identify areas that need more sampling in the future. Third, co-variables used in our models were of different resolution (though they were all finer than the species diversity resolution). For example, soil pH had a significant effect on plant species diversity. However, when this variable was used as an averaged value, its utility may have been reduced since some species are potentially very sensitive to subtle changes in the environment. Fourth, there are uncertainties in areas with few or no saturated grid cells. We attributed this uncertainty to limited sample saturation in the inland areas to draw inferences on the effects of correlated covariables on species diversity. Thus, caution is needed for the interpretation of results in under-sampled areas (Fig. S3, mostly located in the inland areas where population densities were low). In addition, we acknowledge that there are possible other factors that may affect the relationship between wild plant species diversity and SES factors. However, our main purpose in this analysis is to examine whether biodiversity exposure differs by subpopulation groups with different SES factors. It is beyond the scope of the work to examine any causal relationships between the SES and biodiversity. Thus, we did not include other variables in this association analysis. Finally, estimates of the SES indicators, race/ethnicity information, and vulnerable population indices were before 2019, and therefore slightly mismatched with the species diversity indices. Future research is needed to better understand how plant species diversity interacts with environmental factors to further improve modeling efforts and build more precise metrics, as well as incorporate more measurements of biodiversity, such as genetic and ecosystem diversity.

## 5. Conclusion

We developed an analytic framework for mapping continuous taxonomic diversity surfaces in large areas using crowdsourcing data while accounting for critical biases. Such biases included geographic and user selection biases along with the unequal sampling issue that often undermines volunteer-gathered datasets. Our grid-based maps provide the first large-scale perspective on the spatial variation of the wild vascular plant species diversity in 2019–2022. Our results highlight that plant species diversity was disproportionately distributed across socioeconomic lines and race/ethnicity groups in California urban areas. What is more, communities with a higher percentage of children had substantively lower species diversity levels. Our analytic workflow represents a cost-effective way of characterizing biodiversity across large spatial scales and can provide visual diversity patterns and in turn promote greater biodiversity and research investigations on the intersection of biodiversity and health.

## Supplementary Material

2

Li 2023 SoTOTEN. Appendix B

Li 2023 SoTOTEN. Appendix A

## Figures and Tables

**Table 1:** Summary statistics of the wild vascular plant species diversity metrics observed in 841 grid cells, non-optimized metrics from GAM OK, and the optimized metrics for California urban areas.

	Method	Variable	Mean	Median	Minimum	Maximum	Std. dev.

Species richness		Observed metrics for 841 grid cells	147	146	30	378	52
	Non-optimized	Metrics from GAM OK for 4755 grid cells	109	106	39	322	43
	Optimized	Observed + metrics from GAM OK for 4755 grid cells	109	104	30	378	45
Shannon diversity		Observed metrics for 841 grid cells	106	104	21	300	38
	Non-optimized	Metrics from GAM OK for 4755 grid cells	77	75	22	223	33
	Optimized	Observed + metrics from GAM OK for 4755 grid cells	77	74	21	300	34
Simpson diversity		Observed metrics for 841 grid cells	76	73	12	239	30
	Non-optimized	Metrics from GAM OK for 4755 grid cells	55	53	14	182	25
	Optimized	Observed + metrics from GAM OK for 4755 grid cells	55	52	12	239	26

Notes: Non-optimized: metrics from GAM OK without any modifications are referred to as the “non-optimized” models. Optimized: unsaturated grid cells (no data or low completes) with metrics from GAM OK are retained; values in 841 saturated grid cells are replaced with the observed metrics from the iNEXT step. Std. dev.: standard deviation.

**Table 2:** Results of the 10-fold cross-validation.

		Method	Obs. Vs. pred	Prediction errors			
				Root mean squared errors	Relative root mean squared errors (%)	Mean absolute errors	Relative mean absolute errors (%)

Species richness	Training	GLM	0.56	42.73	29.07	33.14	22.54
		GAM	0.78	32.82	22.33	25.44	17.31
	Validation	OK	0.61	40.97	27.87	32.61	22.18
		GLM	0.54	43.61	29.67	33.89	23.05
		GAM	0.64	39.36	26.78	30.50	20.75
		GLM OK	0.54	43.40	29.52	33.73	22.95
		GAM OK	0.64	39.27	26.71	30.43	20.70
Shannon diversity	Training	GLM	0.56	31.81	30.01	24.64	23.25
		GAM	0.79	23.81	22.46	18.32	17.28
	Validation	OK	0.63	29.57	27.90	23.39	22.07
		GLM	0.53	32.62	30.77	25.20	23.77
		GAM	0.66	28.97	27.33	22.35	21.08
		GLM OK	0.54	32.40	30.57	25.03	23.61
		GAM OK	0.66	28.85	27.22	22.25	20.99
Simpson diversity	Training	GLM	0.55	24.82	32.66	19.20	25.26
		GAM	0.77	19.22	25.29	14.67	19.30
	Validation	OK	0.63	22.94	30.18	18.05	23.75
		GLM	0.52	25.45	33.49	19.66	25.87
		GAM	0.64	22.87	30.09	17.59	23.14
		GLM OK	0.53	25.24	33.21	19.49	25.64
		GAM OK	0.64	22.76	29.95	17.49	23.01

Notes: Obs. vs. Pred.: results of Pearson correlation between observed and predicted values.

**Table 3:** Description of the census tract-level plant diversity metrics.

	Disadvantaged communities *n* = 2155	Other communities *n* = 4984	Total *n* = 7139	p-Value

Species richness, mean (SD)	127.5 (34)	146.9 (41.1)	141 (40.1)	<0.001
Shannon diversity, mean (SD)	86.2 (24.9)	101.2 (30.4)	96.7 (29.6)	<0.001
Simpson diversity, mean (SD)	57.1 (17.9)	69.9 (22.7)	66 (22.1)	<0.001

Notes: p-value were from *t*-test to determine the difference in plant species diversity between disadvantaged and non-disadvantaged communities.

**Table 4:** Association between socioeconomic status and plant species diversity metrics in California urban census tracts.

Socioeconomic status indicators	Species richness		Shannon diversity		Simpson diversity	
	Coefficient	95 % CI	Coefficient	95 % CI	Coefficient	95 % CI

Population characteristics score, 0–100	−0.22	−0.26, −0.18	−0.14	−0.17, −0.12	−0.11	−0.13, −0.09
Educational attainment, %	−0.29	−0.34, −0.24	−0.17	−0.21, −0.14	−0.12	−0.15, −0.09
Housing burden, %	−0.06	−0.15, 0.04	−0.06	−0.12, 0.01	−0.07	−0.12, −0.02
Linguistic isolation, %	−0.35	−0.44, −0.27	−0.23	−0.29, −0.17	−0.16	−0.2, −0.11
Poverty, %	−0.13	−0.17, −0.08	−0.09	−0.12, −0.06	−0.08	−0.11, −0.06
Unemployment, %	−0.61	−0.81, −0.4	−0.43	−0.58, −0.29	−0.35	−0.46, −0.25
Non-Hispanic White, %	0.24	0.21, 0.27	0.15	0.13, 0.17	0.11	0.09, 0.12
Hispanic, %	−0.15	−0.18, −0.13	−0.09	−0.11, −0.07	−0.06	−0.08, −0.05
African American, %	−0.15	−0.23, −0.07	−0.13	−0.19, −0.08	−0.12	−0.17, −0.08
Asian American, %	−0.15	−0.2, − 0.1	−0.11	−0.14, −0.07	−0.07	−0.09, −0.04
Native American, %	0.49	−0.7, 1.69	0.54	−0.32, 1.4	0.49	−0.14, 1.12
Multiple races, %	1.25	0.95, 1.55	0.77	0.56, 0.99	0.5	0.34, 0.66
Children, %	−0.79	−0.96, −0.62	−0.44	−0.56, −0.32	−0.27	−0.36, −0.18
Elderly, %	0.12	0.01, 0.23	0.12	0.04, 0.19	0.13	0.07, 0.19

Notes: All GLMM models are adjusted for population density and spatial autocorrelation. CI: confidential interval.

## Data Availability

We have shared the link to download data in the paper

## HIGH LIGHTS

- Developed an analytic framework for mapping plant species diversity using crowdsourced data.
- Estimated wild vascular plant species diversity in large areas at 5 km resolution.
- Revealed an inequitable distribution of plant species diversity by sociodemographic status factors in California.
