# Supplementary material for: Mapping wild vascular plant species diversity in urban areas in California using crowdsourcing data by regression kriging: Examining socioeconomic disparities
Source: Sci Total Environ. Author manuscript; Available in PMC 2024 Dec 20. (PMC10947671; doi:10.1016/j.scitotenv.2023.166995)

**Supplementary Materials**

**TABLE OF CONTENTS**

Contents

Method S0 Description of data filtering process from the iNaturalist platform 2

Table S1 List of co-variables in regression kriging models 3

Table S2 Selected indicators, description, and year of data source from CalEnviroScreen4.0 tool 4

Table S3 Final Model of the GLM and GAM 5

Table S4 Characterization of the population characteristics in California urban census tracts 6

Table S5 Pearson correlation coefficients of indicators from CalEnviroScreen4.0 database with census tract-level wild vascular plant diversity metrics in urban California (N=6689) 6

Table S6 Association between socioeconomic status and plant species diversity metrics in California urban census tracts (full model) 7

Table S7 Association between sociodemographic status and plant species diversity metrics in California urban census tracts after adjusting for NDVI (single factor model) 7

Figure S1 The 80% sample coverage-based wild vascular plant species Shannon diversity metrics (A), and species Simpson diversity metrics (B) within the study region 8

Figure S2 Spatial distribution of wild vascular plant species Shannon diversity metrics (A), and Simpson diversity metrics (B) in California urban areas (2019-2022) (resolution: 5 km) 10

Figure S3 Uncertainty in extrapolated vascular plant species richness, species Shannon diversity and species Simpson diversity metrics from the Generalized Additive Model with Ordinary kriging 13

Figure S4 Spatial pattern of census tract-level wild vascular plant species richness metrics(A), species Shannon diversity metrics (B), and species Simpson diversity metrics (C) in California urban areas 16

Figure S5 Spatial pattern of census tract-level population density 19

Figure S6 Spatial pattern of census tract-level other/multiple races proportion 20

# Method S0 Description of data filtering process from the iNaturalist platform

iNaturalist provides a platform for citizen scientists to record and share biodiversity information. It has an organism occurrence recording tool and a crowdsourced species identification system. iNaturalist is primarily focused on observing wild organisms. It shares its data with Global Biodiversity Information Facility through weekly automatic exports of research-grade observations that meet licensing requirements (More details on licenses: https://www.gbif.org/dataset/50c9509d-22c7-4a22-a47d-8c48425ef4a7#description). The research-grade observation data is free and publicly accessible.

Data entry and quality control primarily depended on the input provided by users of the iNaturalist platform, who submit their observations and identify observations submitted by others. There are two stages: 1) User submission: When users upload a new observation, they are asked to specify whether the observed organism is wild/naturalized or captive/cultivated. The observation will then only be considered a verifiable observation if it has a date, georeference, photo or audio identifier, and is wild (not cultivated). 2) Data Quality Assessment: Other iNaturalist users can agree or disagree with the verifiable observations such as taxon and wild/cultivated attributes. If more than 2/3 of a minimum of three users agree with the identification of the photographed plants with species level, observations will be considered “Research Grade”.

Plants photographed in private gardens will not be marked as wild and will be excluded from the research-grade dataset. Nevertheless, the species' seeds that escaped from gardens and managed to grow by themselves outside the gardens are considered wild. If citizen scientists are not sure about whether an observation is cultivated or wild, they can add notes and reserve the “wild” labeling for those observations. Those observations will go through the data quality assessment by the users and the system. The system will automatically vote that the observation is not wild/naturalized if there are at least 10 other observations of a genus or lower in the smallest county-, state-, or country-equivalent place that contains this observation and 80% or more of those observations have been marked as not wild or naturalized. If more than 2/3 of a minimum of three votes (from users and the system) agree on the identification, then the observation will remain research-grade.

# Table S1 List of co-variables in regression kriging models

| **Variables** | **Source** | **Data Source** |
| --- | --- | --- |
| Average annual temperature | Mean annual temperature per grid cell (°F) | 30-year annual mean temperature (1991-2020). Dataset: https://prism.oregonstate.edu/normals/ (resolution: 800m) |
| Average annual precipitation | Mean annual precipitation per grid cell (inch) | 30-year annual mean precipitation (Inch) (1991-2020). Dataset: https://prism.oregonstate.edu/normals/ (resolution: 800m) |
| Average elevation | Average elevation per grid cell (m) | The global multi-resolution Terrain elevation data (m) (2010). https://www.usgs.gov/centers/eros/science/usgs-eros-archive-digital-elevation-global-multi-resolution-terrain-elevation (resolution: 7.5 arcsec (c.30m)) |
| Tree Canopy | Average percentage of tree canopy cover per grid cell | National Land Cover Database 2016 (percentage) - Tree Canopy (NLCD2016). https://www.mrlc.gov/data/references/national-land-cover-database-2016-tree-canopy-nlcd2016 (resolution: 30m) |
| Greenness (NDVI) | Mean Normalized Difference Vegetation Index per grid cell | MODIS Vegetation Index Products (2020). https://modis.gsfc.nasa.gov/data/dataprod/mod13.php (resolution: 250m) |
| Percentage of water | Percentage of water per grid cell | NCLD 2019 Land Cover (CONUS). https://www.mrlc.gov/data/nlcd-2019-land-cover-conus (resolution: 30m) |
| Percentage of wetlands | Percentage of wetlands per grid cell |  |
| Percentage of developed open space | Percentage of urban open space per grid cell |  |
| Percentage of herbaceous | Percentage of herbaceous per grid cell |  |
| Percentage of shrubland | Percentage of shrubland per grid cell |  |
| Percentage of Planted | Percentage of planted area per grid cell |  |
| Road Density | Percentage of primary, secondary, tertiary, and thinned road density per grid cell | NCLD 2019 Developed Imperviousness Descriptor (CONUS). https://www.mrlc.gov/data/nlcd-2019-developed-imperviousness-descriptor-conus (resolution: 30m) |
| Actual evapotranspiration | Average annual actual evapotranspiration | 2011 California Basin Characterization Model (BCM) Downscaled Climate and Hydrology 30-year Summaries. http://climate.calcommons.org/dataset/10 (resolution: 270 m) |
| Potential evapotranspiration | Average annual potential evapotranspiration | Average Annual Evapo-Transpiration (1970-2000). https://www.nature.com/articles/s41597-022-01493-1#data-availability (resolution: 30 arcsec (c. 1 km × 1 km)) |
| Calcium carbonate | Average calcium carbonate per grid cell (kg/m2) | Soil Survey Geographic Database. https://casoilresource.lawr.ucdavis.edu/soil-properties/download.php (resolution: 800m) |
| Cation exchange cap | Average cation exchange capacity per grid cell (cmol/kg) |  |
| Electrical conductivity | Average electrical conductivity per grid cell (dS/m) |  |
| pH | Average pH per grid cell (pH) |  |
| Sodium adsorption ratio | Average sodium adsorption ratio per grid cell |  |
| Soil organic matter | Average organic matter per grid cell (kg/m2) |  |
| Available water holding capacity | Average available water holding capacity per grid cell (cm) |  |
| Bulk density, 1/3 bar | Average bulk density per grid cell (g/cm3) |  |
| Longitude | Centroids of each grid cell | - |
| Latitude |  |  |

# Table S2 Selected indicators, description, and year of data source from CalEnviroScreen4.0 tool

| **Categories** | **Variables** | **Year** | **Description** |
| --- | --- | --- | --- |
| Socioeconomic Factor indicators (%) | Educational Attainment | 2015-2019 | Percent of the population over age 25 with less than a high school education |
|  | Housing Burden | 2013-2017 | Percent of households in a census tract that are both low income and severely burdened by housing costs |
|  | Linguistic Isolation | 2015-2019 | Percent of limited English-speaking households |
|  | Poverty | 2015-2019 | Percent of the population living below two times the federal poverty level |
|  | Unemployment | 2015-2019 | Percent of the population over the age of 16 that is unemployed and eligible for the labor force |
| Summary Indicators | Population Characteristics Score | ·· | Derived from the average percentiles for the three Sensitive populations indicators and the five socioeconomic factors indicators and ranged from 0.1-10 |
|  | CalEnviroScreen4.0 Score | ·· | Pollution score multiplied by population characteristics score. Higher CES4.0 scores have relatively high pollution burdens and population sensitivities |
| Racial/Ethnic Indicators (%) | Hispanic | 2019 | Percent per Census Tract of those who identify as Hispanic or Latino |
|  | White | 2019 | Percent per Census Tract of those who identify as non-Hispanic white |
|  | African American | 2019 | Percent per Census Tract of those who identify as non-Hispanic African American or black |
|  | Native American | 2019 | Percent per Census Tract of those who identify as non-Hispanic Native American |
|  | Asian American | 2019 | Percent per Census Tract of those who identify as non-Hispanic Asian or Pacific Islander |
|  | Other/Multiple | 2019 | Percent per Census Tract of those who identify as non-Hispanic "other" or as multiple races |
| Sensitive population Indicators | Asthma (per 10,000) | 2015-2017 | Age-adjusted rate of Emergency department (ED) visits for asthma |
|  | Low Birth Weight (%) | 2009-2015 | Percent low birth weight |
|  | Cardiovascular Disease (per 10,000) | 2015-2017 | Age-adjusted rate of ED visits for acute myocardial infarction |
| Vulnerable population Indicators | Children < 10 years (%) | 2019 | Percent per Census Tract of children under 10 years old |
|  | Elderly > 64 years (%) | 2019 | Percent per Census Tract of elderly 65 years and older |

# Table S3 Final Model of the GLM and GAM

| **Covariables** | **Species Richness** | | | | **Shannon Diversity** | | | | **Simpson Diversity** | | | |
| --- | --- | --- | --- | --- | --- | --- | --- | --- | --- | --- | --- | --- |
|  | **GLM** | | **GAM** | | **GLM** | | **GAM** | | **GLM** | | **GAM** | |
|  | **Model** | **Significance** | **Model** | **Significance** | **Model** | **Significance** | **Model** | **Significance** | **Model** | **Significance** | **Model** | **Significance** |
| Longitude | x | ** | x | *** | x | *** | x | *** | x | ** | x | *** |
| Latitude | - | - |  |  | - | - |  |  | - | - |  |  |
| Temperature | x | ** | x | >0.05 | x | >0.05 | x | * | x | >0.05 | x | * |
| Precipitation | x | *** | x | * | x | *** | x | * | x | *** | x | * |
| Elevation | - | - | x | >0.05 | x | >0.05 | x | >0.05 | x | * | x | >0.05 |
| Potential Evapotranspiration | x | *** | x | *** | x | *** | x | *** | x | *** | x | *** |
| Tree Canopy | x | *** | x | ** | x | *** | x | ** | x | *** | x | *** |
| NDVI | - | - | - | - | - | - | - | - | - | - | x | * |
| Water | x | *** | x | *** | x | *** | x | *** | x | *** | x | *** |
| Herbaceous | x | * | x | >0.05 | x | *** | x | >0.05 | x | *** | x | >0.05 |
| Planted | x | *** | x | * | x | *** | x | ** | x | *** | x | ** |
| Developed Open Space | x | >0.05 | x | * | x | >0.05 | x | * | x | >0.05 | x | >0.05 |
| Primary Road Density | x | * | x | * | x | ** | x | * | x | * | x | ** |
| Secondary Road Density | x | >0.05 | x | *** | - | - | x | *** | - | - | x | >0.05 |
| Tertiary Road Density | x | >0.05 | x | * | - | - | x | >0.05 | - | - | x | >0.05 |
| Calcium Carbonate | x | ** | x | >0.05 | x | *** | x | ** | x | *** | x | * |
| Cation Exchange Cap | x | ** | - | - | - | - | - | - | - | - | - | - |
| Electrical Conductivity | x | ** | x | >0.05 | x | *** | - | - | x | *** | - | - |
| pH | x | >0.05 | x | ** | - | - | x | ** | - | - | x | ** |
| Sodium Adsorption Ratio | x | ** | x | >0.05 | x | *** | - | - | x | *** | - | - |
| Bulk Density | - | - | x | * | x | ** | x | * | x | ** | x | * |
| Organic Matter | x | * | x | >0.05 | x | * | x | * | x | * | x | * |
| Deviance Explained (%) | 34.6 | | 61 | | 33.9 | | 62.8 | | 32.9 | | 59.5 | |
| 10-fold Cross Validation  (Deviance Explained (%)) | 34.8 | | 60.1 | | 34.1 | | 61.6 | | 33.1 | | 58 | |

Notes: x indicates the variable included in the model; - means the variable is not included in the model. * <0.05; ** <0.1, ***<0.01.

# Table S4 Characterization of the population characteristics in California urban census tracts

| **Characteristics** | **Disadvantaged communities n=2155** | **Other communities n=4984** | **Total n=7139** |
| --- | --- | --- | --- |
| **Socioeconomic status** |  |  |  |
| Educational attainment, % | 31.89 (13.24) | 11.3 (10.16) | 17.54 (14.65) |
| Linguistic Isolation, % | 16.6 (10.19) | 7.21 (7.39) | 10.06 (9.4) |
| Poverty, % | 48.16 (14.85) | 23.5 (14.27) | 30.95 (18.36) |
| Unemployment, % | 8.19 (4.27) | 5.26 (3.04) | 6.15 (3.71) |
| Housing Burden, % | 25.07 (8.05) | 16.15 (7.05) | 18.85 (8.43) |
| **Race/Ethnicity** |  |  |  |
| Hispanic, % | 63.27 (22.36) | 28.17 (20.31) | 38.77 (26.43) |
| White, % | 15.16 (14.78) | 45.57 (23.02) | 36.39 (25.12) |
| African American, % | 8.93 (11.56) | 4.71 (7.06) | 5.98 (8.88) |
| Native American, % | 0.35 (3.1) | 0.28 (0.85) | 0.30 (1.85) |
| Asian American, % | 10.27 (12.28) | 17.44 (16.98) | 15.28 (16.5) |
| Other/multiple races, % | 2.02 (2.12) | 3.84 (2.37) | 3.29 (2.45) |
| **Vulnerable Population Indicators** |  |  |  |
| Children < 10 years, % | 13.99 (4.33) | 11.24 (4.19) | 12.07 (4.42) |
| Elderly > 64 years, % | 10.84 (5.22) | 15.87 (8.48) | 14.35 (7.99) |
| **Population Characteristics Score, 0-10** | 7.43 (1.14) | 4.22 (1.74) | 5.19 (2.16) |

# Table S5 Pearson correlation coefficients of indicators from CalEnviroScreen4.0 database with census tract-level wild vascular plant diversity metrics in urban California (N=6689)

| **Category** | **Indicators** | **Species Richness** | **Shannon Diversity** | **Simpson Diversity** |
| --- | --- | --- | --- | --- |
| **Socioeconomic factors** | Educational Attainment | **-0.25** | **-0.25** | **-0.27** |
|  | Housing Burden | -0.05 | -0.07 | -0.1 |
|  | Linguistic Isolation | -0.13 | -0.14 | -0.17 |
|  | Poverty | **-0.23** | **-0.24** | **-0.26** |
|  | Unemployment | **-0.25** | **-0.25** | **-0.25** |
| **Race/Ethnicity** | Hispanic | **-0.29** | **-0.29** | **-0.3** |
|  | White | **0.25** | **0.26** | **0.3** |
|  | African American | -0.07 | -0.09 | -0.11 |
|  | Native American | -0.02 | -0.01 | -0.002 |
|  | Asian American | 0.1 | 0.08 | 0.06 |
|  | Other/Multiple races | **0.21** | **0.21** | **0.21** |
| **Vulnerable population indicators** | Children < 10 years | **-0.25** | **-0.23** | **-0.21** |
|  | Elderly > 64 years | 0.1 | 0.11 | 0.14 |
| **Summary Indicator** | Population Characteristics Score, 0-10 | **-0.31** | **-0.32** | **-0.35** |

Notes: **Bold** indicates correlation coefficients > 0.2 and statistical significance (p<0.05).

**Table S5** presents correlation analyses and shows species diversity metrics to be negatively correlated with all SES factors, with correlations being most pronounced for educational attainment (e.g., r = -0.27 for Simpson diversity)., followed by unemployment rate, and poverty. A higher diversity level was correlated with a higher percentage of non-Hispanic Whites (r = 0.25 to 0.3). Census tracts with higher proportions of Hispanic/Latino residents had lower species diversity levels (r = -0.3 to -0.29). The population characteristics score (where higher values indicate a higher average percentile of sensitive populations) showed a significantly negative correlation (r= -0.35 to -0.31) with species diversity metrics.

# Table S6 Association between socioeconomic status and plant species diversity metrics in California urban census tracts (full model)

| **Socioeconomic status indicators** | **Species richness** | | **Shannon Diversity** | | **Simpson Diversity** | |
| --- | --- | --- | --- | --- | --- | --- |
|  | **coefficient** | **95% CI** | **coefficient** | **95% CI** | **coefficient** | **95% CI** |
| Educational Attainment, % | -0.23 | -0.3, -0.17 | -0.12 | -0.17, -0.07 | -0.08 | -0.12, -0.05 |
| Linguistic Isolation, % | -0.09 | -0.2, 0.01 | -0.09 | -0.17 -0.01 | -0.06 | -0.11, 0.001 |
| Unemployment, % | -0.25 | -0.46, -0.03 | -0.23 | -0.38, -0.07 | -0.22 | -0.33, -0.1 |

Notes: All three GLMM full models are further adjusted for population density and spatial autocorrelation. CI: confidential interval.

# Table S7 Association between sociodemographic status and plant species diversity metrics in California urban census tracts after adjusting for NDVI (single factor model)

| **Socioeconomic status indicators** | **Species richness** | | **Shannon Diversity** | | **Simpson Diversity** | |
| --- | --- | --- | --- | --- | --- | --- |
|  | **coefficient** | **95% CI** | **coefficient** | **95% CI** | **coefficient** | **95% CI** |
| Population Characteristics Score, 0-10 | -0.17 | -0.21, -0.13 | -0.09 | -0.12, -0.06 | -0.06 | -0.08, -0.03 |
| Educational Attainment, % | -0.24 | -0.29, -0.18 | -0.11 | -0.15, -0.07 | -0.06 | -0.09, -0.03 |
| Housing Burden, % | 0.09 | -0.01, 0.18 | 0.07 | 0.001, 0.14 | 0.05 | -0.001, 0.1 |
| Linguistic Isolation, % | -0.27 | -0.36, -0.18 | -0.15 | -0.21, -0.09 | -0.08 | -0.13, -0.03 |
| Poverty, % | -0.06 | -0.1, -0.01 | -0.02 | -0.05, 0.02 | -0.01 | -0.04, 0.01 |
| Unemployment, % | -0.46 | -0.66, -0.25 | -0.29 | -0.44, -0.15 | -0.22 | -0.33, -0.11 |
| non-Hispanic White, % | 0.21 | 0.18, 0.24 | 0.12 | 0.1, 0.14 | 0.07 | 0.06, 0.09 |
| Hispanic, % | -0.12 | -0.15, -0.09 | -0.06 | -0.08, -0.03 | -0.03 | -0.04, -0.01 |
| African American, % | -0.1 | -0.18, -0.02 | -0.09 | -0.14, -0.03 | -0.08 | -0.12, -0.03 |
| Asian American, % | -0.16 | -0.21, -0.11 | -0.11 | -0.15, -0.08 | -0.07 | -0.1, -0.05 |
| Native American, % | 0.6 | -0.59, 1.79 | 0.64 | -0.22, 1.49 | 0.58 | -0.04, 1.21 |
| Multiple race, % | 1.08 | 0.78, 1.39 | 0.61 | 0.4, 0.83 | 0.34 | 0.18, 0.5 |
| Children, % | -0.7 | -0.87, -0.53 | -0.36 | -0.48, -0.24 | -0.19 | -0.28, -0.1 |
| Elderly, % | -0.01 | -0.13, 0.1 | -0.01 | -0.09, 0.07 | 0.02 | -0.04, 0.08 |

Notes: All GLMM models are adjusted for population density and NDVI. CI: confidential interval

# Figure S1 The 80% sample coverage-based wild vascular plant species Shannon diversity metrics (A), and species Simpson diversity metrics (B) within the study region

**A**





**B**





# Figure S2 Spatial distribution of wild vascular plant species Shannon diversity metrics (A), and Simpson diversity metrics (B) in California urban areas (2019-2022) (resolution: 5 km)

**A**

**

**

**B**





Notes: For visual purposes, we cut off the grid cells outside the California land boundary. This results in a discrepancy in the minimums when comparing Figure S2 with the optimized version of metrics in Table 1.

# Figure S3 Uncertainty in extrapolated vascular plant species richness, species Shannon diversity and species Simpson diversity metrics from the Generalized Additive Model with Ordinary kriging

A



B





C





# Figure S4 Spatial pattern of census tract-level wild vascular plant species richness metrics(A), species Shannon diversity metrics (B), and species Simpson diversity metrics (C) in California urban areas

**A**


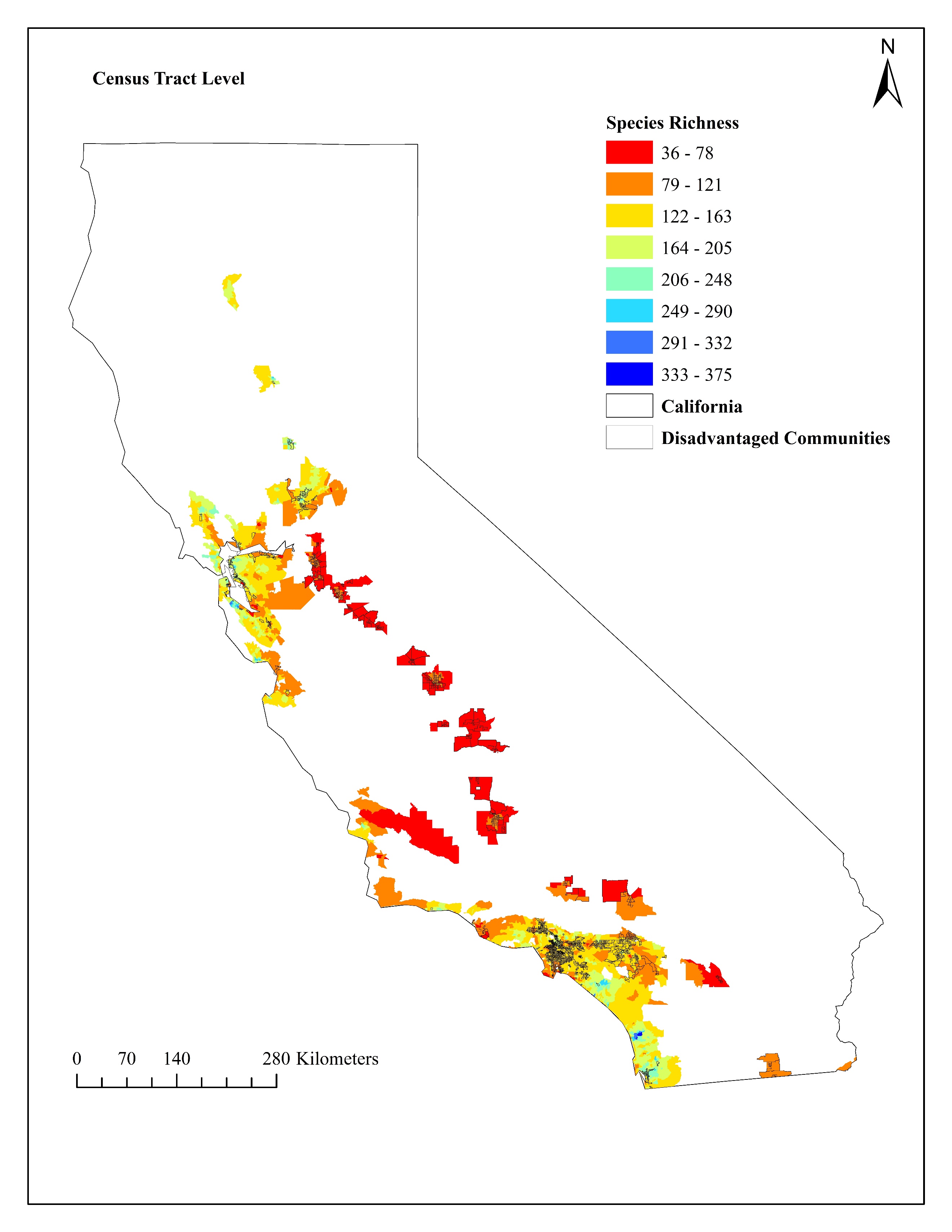


**B**

**

**

**C**


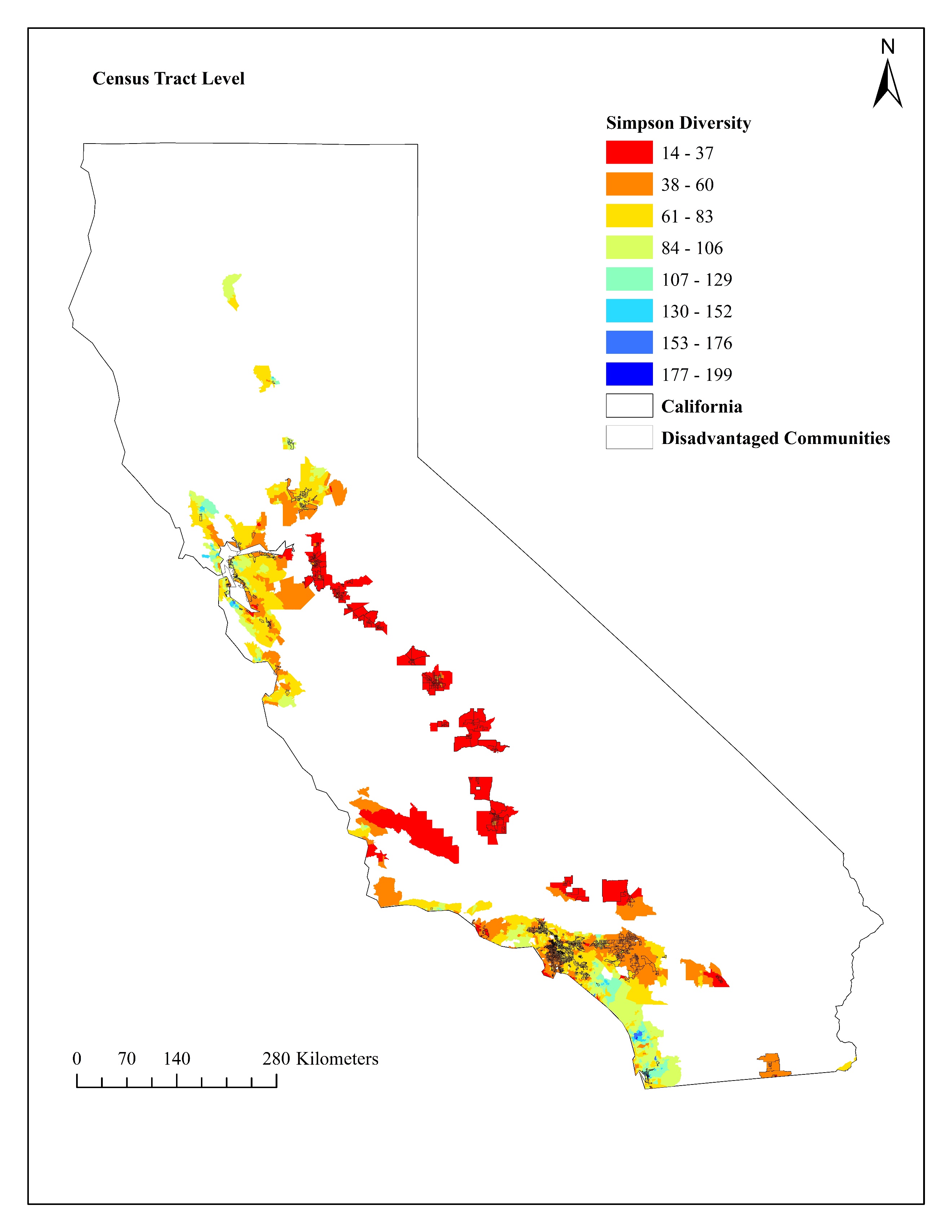
Notes: The black line highlights the location of disadvantaged communities.

# Figure S5 Spatial pattern of census tract-level population density

**

**

# Figure S6 Spatial pattern of census tract-level other/multiple races proportion


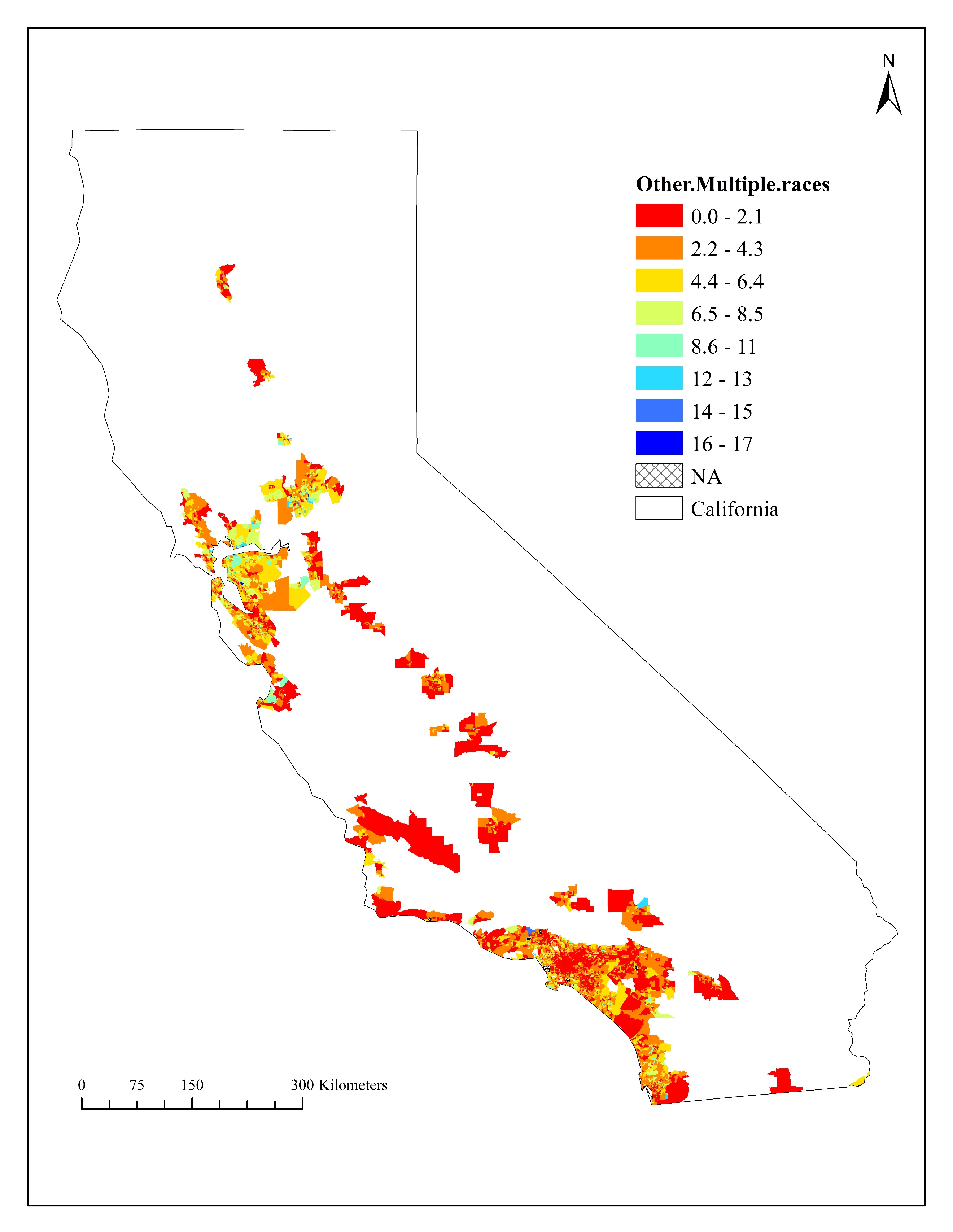

Supplement: 2 [file NIHMS1967189-supplement-2.docx]
